# Issues in cytomegalovirus chronic infection in HIV patients

**DOI:** 10.1186/1471-2334-14-S4-P40

**Published:** 2014-05-29

**Authors:** Arbune Manuela, Miruna Drăgănescu, Dana Tutunaru, Costinela Georgescu

**Affiliations:** 1 “Dunărea de Jos” University, Galați, Romania

## 

The prevalence of cytomegalovirus (CMV) in HIV infected patients is 90%. Both CMV and HIV are involved in the pathogenesis of metabolic and cardiovascular diseases.

The study retrospectively evaluated the correlation of CMV infection with lipodystrophy (LD) and hypertension (HTA) in HIV patients who have been beneficiaries of medical care in Galați, during 2013. The evolution of anthropometric parameters, CDC-HIV categories, the immunity by LyCD4, the category of detectable or undetectable HIV-RNA, the categories high/low for values of cholesterol, triglycerides, glucose, fibrinogen and for serological CMV-IgG (ELISA), were collected from the records of 165 patients. CMV-IgG titers were converted in high or low dichotomous variables, referring to the quartile (Q) 3. Statistical analysis (XL-Stat) evaluated the correlations between groups, with χ^2^ test for dichotomous variables and Student’s t test for continuous variables.

The characteristics of patients are: median age 25 years [20;50]; men sex 53.7%; rural area 50.3%; low literacy (<4 years) 25%; smokers 58%; co-infection with HBV 28.5%; median length of HIV diagnosis 12 years; AIDS category 74%; current antiretroviral treatment 95%, with average 2 [0;8] previous combinations; median nadir LyCD4=144/cmm, median current LyCD4=507/cmm, undetectable HIV-RNA (<400 copies/mL) 69%. The prevalence of positive CMV-IgG is 87% and 25.5% of the values are over Q3. Hypertension was found in 9.7% and LD in 48% of patients, specifying 36% atrophy (LA), 6% hypertrophy (LH) and 6% mixed lipodystrophy (ML). High values of CMV-IgG were associated with severe immunosuppression of nadir LyCD4 (p=0.002), high fibrinogen level (p<0.001), HTA (p=0.032) and ML pattern (p<0.001). There was no correlation of CMV-IgG with LA, LH, HIV-RNA, cholesterol, triglycerides and glucose.

The levels of CMV-IgG should be monitored in HIV patients as a risk factor for hypertension and mixed lipodystrophy in order to allow early interventions for management of these co-morbidities.

